# 3D Molded Inserts Fabricated Using a Self-Nanoemulsifying Drug Delivery System (SNEDDS) to Increase Voriconazole and Diclofenac Permeation for the Treatment of Corneal Keratitis

**DOI:** 10.3390/polym18121517

**Published:** 2026-06-18

**Authors:** Ahmed Almotairy

**Affiliations:** 1 Department of Pharmaceutics and Pharmaceutical Industries, College of Pharmacy, Taibah University, Madinah 42353, Saudi Arabia; amrmutairi@taibahu.edu.sa; Tel.: +966-506033113; 2 Health and Life Research Center, Taibah University, Madinah 42353, Saudi Arabia

**Keywords:** voriconazole, ocular insert, corneal infection, self-nanoemulsifying drug delivery systems, lipid-based nanoparticle, soluplus

## Abstract

*Acanthamoeba* keratitis (AK) and mycotic keratitis (MK) are severe infectious diseases of the cornea. A major challenge to treatment is the poor bioavailability of conventional eye drop formulations, resulting in significant loss of the therapeutic compound. The present study describes the preparation of self-nanoemulsifying drug delivery systems (SNEDDSs) utilizing 3D-molded inserts as a vehicle for the co-delivery of voriconazole (VOR) and diclofenac sodium (DIC). Characterization of the SNEDDS and the 3D-molded inserts involved analysis of droplet size, polydispersity index (PDI), and zeta potential (ZP), followed by an ex vivo transcorneal permeation. Careful optimization of the SNEDDS composition was fundamental for obtaining a nanoemulsion with a droplet size under 100 nm, a PDI below 0.35, and a variable ZP (ranging from −14.6 ± 0.1 to 16.3 ± 2.4 mV). These properties facilitated a marked improvement in voriconazole’s solubility and subsequent transcorneal permeability. The investigation of the inserts revealed that the in vitro drug release could be tailored for immediate release or extended release. Moreover, ex vivo permeation studies demonstrated that the inserts delivered voriconazole to the cornea at concentrations more than the minimum inhibitory concentration. This work successfully demonstrates the formulation of a patient-centric, personalized drug delivery platform for the treatment of AK and MK.

## 1. Introduction

*Acanthamoeba* spp. are free-living amoebae that thrive in various environments. *Acanthamoeba* spp. can cause sinus infections, cutaneous lesions, and *Acanthamoeba* keratitis (AK), a vision-threatening condition characterized by inflammation of the cornea that can lead to blindness [1]. On the other hand, fungi, particularly *Candida* spp., which are naturally present on the human mucosal surface, can cause mycotic keratitis (MK) [2]. Majority of AK and MK cases are related to contact lens wearers, as contact lens wear may facilitate the direct inoculation of amoebae and fungi [3]. Corneal infections are treated with eye drops containing a combination of chlorhexidine and polyhexamethylene biguanide, Brolene or hexamidine. Chlorhexidine (0.2%) and polyhexamethylene biguanide (0.02%) are effective at low concentrations, even though the former is not toxic to corneal epithelial cells, while the latter is toxic [4,5,6,7]. These combination eye drops must be administered every hour for 3 days and then every 3 h for an additional 3–4 weeks [8]. Importantly, however, some patients exhibit resistance to chlorhexidine usage and need a combination of other antimicrobial drugs. The primary drugs used in these cases include amphotericin B, pentamidine, rifampicin, flucytosine, and others. Additionally, azole antifungal drugs including voriconazole can be used either orally or topically. If anterior chamber inflammation is present, the concomitant use of corticosteroids or nonsteroidal anti-inflammatory drugs (NSAIDs) is needed. However, the primary challenge in treating AK and MK is the poor transcorneal permeation of the drug into the site of infection [9,10,11,12]. Diclofenac sodium is an NSAID that nonselectively, reversibly and competitively inhibits cyclooxygenase (COX) enzymes. COX inhibition prevents the conversion of arachidonic acid into prostaglandins, which are involved in pain, inflammation and fever. Currently, 0.1% diclofenac eye drops are prescribed for ocular inflammation [13]. To alleviate inflammation and pain in the cornea associated with AK or MK, diclofenac may be used in combination with an amoebicidal or fungicidal agent. Although the eye is easily accessible for drug administration, its complicated structure makes drug delivery challenging. Additionally, the drugs used for AK and MK treatment have high molecular weights and low water solubilities, hindering their passage through ocular barriers to reach the site of infection.

Voriconazole, a second-generation triazole, is commonly used for the treatment of keratitis. Voriconazole disrupts the integrity of the fungal cell membrane by binding to the cytochrome P-450 enzyme lanosterol 14-α-demethylase, resulting in enhanced cell membrane permeability that impairs cellular function and halts growth [14]. Voriconazole has a wide spectrum of activity against amoebas and fungal species associated with keratitis [15]. However, it is a BCS class II drug owing to its poor water solubility and high permeability [16]. Consequently, reaching a therapeutic concentration through conventional administration routes may pose challenges. Furthermore, visual disturbances, photopsia, and hepatotoxicity are the known systemic adverse effects of using voriconazole [17,18].

Topical delivery of a sustained-release formulation of VOR could improve outcomes while minimizing adverse effects. Ocular inserts are polymeric matrices designed to be inserted into the eye cavity to reduce dosing frequency and prolong drug delivery, thus decreasing toxicity and systemic exposure. Self-nanoemulsifying drug delivery systems (SNEDDSs) are considered novel systems for addressing the problems of formulating poorly water-soluble drugs [19,20]. SNEDDSs are isotropic mixtures of oils, surfactants, and cosurfactants that produce oil-in-water (O/W) nanoemulsions upon dilution with aqueous media such as tear fluids. Lipophilic drugs, such as VOR, are dissolved in the small droplets of the generated nanoemulsion, which increases their absorption due to the increased surface area [21].

The aim of this project was to formulate an immediate and extended-release VOR formulation and a diclofenac sodium (DIC)-loaded SNEDDS to produce ocular inserts fabricated by 3D-printed mold. Soluplus^®^ was chosen to produce the inserts because of its hydrophilicity, suitability for insert formation, biocompatibility, and biodegradability. Soluplus^®^ has been used extensively in the literature with ocular formulations [22,23]. Additionally, Eudragit^®^ RS100 and Compritol^®^ 888 ATO were also chosen to investigate their ability to produce sustained-release inserts on the basis of literature reports [24,25,26,27]. To the best of our knowledge, this is the first study to generate a patient-centric, dual-drug VOR:DIC-loaded SNEDDS for immediate and extended-release ocular inserts for the management or treatment of *Acanthamoeba* keratitis and mycotic keratitis.

## 2. Materials and Methods

### 2.1. Materials

Voriconazole (assay ≥ 98%) was a gift from Neuland laboratories (Hyderebad, India). Soluplus^®^ (polyvinyl caprolactam−polyvinyl acetate−polyethylene glycol graft copolymer) was a gift from BASF (Ludwigshafen, Germany). Glyceryl dibehenate (Compritol^®^ 888 ATO) and Eudragit^®^ RS100 were gifts from UFC Biotechnology (Amherst, NY, USA). Diclofenac sodium salt (assay ≥ 98%), isopropyl myristate (IPM), polyoxyethylene (20) sorbitan monooleate (Tween 80), and propylene glycol (PG) were obtained from Sigma–Aldrich Chemie-GmbH (Steinheim, Germany). Ultrapure water was obtained using a Millipore water filtration system (Bedford, MA, USA). Other materials and solvents were of pharmaceutical or analytical grade and were utilized as received.

### 2.2. Methods

#### 2.2.1. High-Performance Liquid Chromatography (HPLC)

VOR and DIC were quantified by a validated HPLC assay using a Thermo BDS Hypersil C18 column (Thermo Fisher, Waltham, MA, USA) (150 mm × 4.6 mm, 5 μm) at ambient temperature. The mobile phase was a mixture of methanol and water (30:70, *v*/*v*) delivered at a flow rate of 1.0 mL/min, with UV detection at 256 and 276 nm for voriconazole and diclofenac sodium, respectively [28,29].

#### 2.2.2. Preparation of the SNEDDS and Formulations

SNEDDS were prepared by mixing 11.88% (*w*/*w*) IPM (oil), 31.55% (*w*/*w*) PG (cosolvent), and 56.21% (*w*/*w*) Tween 80 (surfactant) using a magnetic stirrer at 37 ± 0.5 °C until a transparent solution was obtained [28,30]. Subsequently, 0.24% (*w*/*w*) voriconazole and 0.12% (*w*/*w*) diclofenac sodium were introduced to the solution with stirring until both drugs had dissolved [29].

The SNEDDS (400 mg) was transferred to a 20 mL scintillation glass vial, and 600 mg of Soluplus^®^ (F-SOL) was added with thorough mixing using a magnetic stirrer at 115 ± 0.5 °C until complete melting was achieved. The other formulations were prepared in a similar manner, with the ratio of Soluplus^®^ to Eudragit^®^ RS100 (F-EUD) or Compritol^®^ 888 ATO (F-COM) maintained at 3:1. All prepared formulations were poured into the 3D-printed mold (≈20 inserts) and stored in a refrigerator for 60 min. Any excess material was removed from the mold by scraping with a blade to ensure that the outer surfaces of the inserts were uniform.

#### 2.2.3. 3D-Printed Mold for Ocular Inserting

A mold consisting of multiple rounded, open rectangular cavities with dimensions of 1 mm in length, 2 mm in width, and 1 mm in thickness was designed using Autodesk Tinkercad software (San Francisco, CA, USA). An STL file was generated and then formatted into gcode format using Ultimaker CURA software (Ultimaker, Geldermalsen, The Netherlands). The mold was printed using an AnkerMake M5C FDM printer (AnkerMake, Changsha, China) with poly(lactic acid) (PLA) as the material using the following parameters: the build plate temperature was 70 °C, the printing temperature was 220 °C, the print speed was 50 mm/s, and the traveling speed was 250 mm/s. The print quality parameters were set as a rectilinear infill pattern with 100% infill and 0.2 mm for the layer height that described the thickness of each layer.

#### 2.2.4. Fourier Transform Infrared (FTIR) Spectroscopy

FTIR spectra were acquired in the range of 4000–400 cm^−1^ using an IRAffinity-1S spectrometer (Shimadzu, Milton Keynes, UK). The bench was equipped with a QUEST ATR accessory (Specac, Orpington, UK) fitted with a single-reflection monolithic diamond ATR crystal. FTIR spectroscopy was employed to determine the molecular interactions in the samples (drugs, excipients, and formulated inserts).

#### 2.2.5. Differential Scanning Calorimetry (DSC)

Drugs, excipients, and formulated inserts’ thermal behaviors were studied by a Netzsch DSC (Netzsch F3 Maia^®^, Netzsch, Selb, Germany) calibrated with indium. The samples (4–7.9 mg) were added to aluminum pans and sealed with an aluminum lid. Then, the samples were subjected to heating from 25 °C to 200 °C at a rate of 10 °C/minute, while maintaining a flow rate of 50 mL/minute in a dry nitrogen atmosphere. An empty aluminum pan was used as a reference.

#### 2.2.6. Drug Content

An accurately weighed sample containing equivalent amounts of drugs (*n* = 3) was completely dissolved in 3 mL of methanol. The solution was sonicated for 10 min and centrifuged at 6000 rpm for 10 min. The supernatants were then properly diluted and analyzed using HPLC.

#### 2.2.7. Thickness, Weight, and Appearance

The appearance and thickness of the inserts were visually inspected and measured using an Adoric digital caliper (0–150 mm). Each insert was individually weighed on a calibrated analytical balance (Adam). All measurements were performed in triplicate.

#### 2.2.8. Assessment of Droplet Size (DS), Polydispersity Index (PDI), and Zeta Potential (ZP)

The DS, PDI, and ZP of the inserts were measured using a Microtrac S3500 (Microtrac Inc., Montgomeryville, PA, USA) at 25 °C. To avoid the influence of multiple scattering, the measurements were performed in triplicate after dilution with double-distilled water (1:100, *v*/*v*).

#### 2.2.9. Surface pH

The inserts were immersed in 5.0 mL of isotonic phosphate-buffered saline (IPBS; pH 7.4) in glass scintillation vials, and the pH was measured using a pH meter calibrated with various buffers with known pH values (4.01, 7.00, and 10.01). Each pH measurement was conducted in triplicate.

#### 2.2.10. In Vitro Release Study

Inserts were accurately weighed and positioned inside a 20 mL scintillation glass vial (*n* = 3). After that, a mesh was added above the inserts, and a magnetic stirrer was placed above the mesh screen. A 10 mL volume of PBS (pH 7.4) was introduced to the vials and maintained at a temperature of 34 ± 0.2 °C under constant stirring. At 5, 10, 15, 30, 60, 120, 240, and 360 min, aliquots of 0.5 mL were withdrawn and replaced with the exact volume using fresh medium to maintain a constant volume [31]. The concentrations of VOR and DIC in all collected aliquots were analyzed using HPLC.

#### 2.2.11. Swelling Behavior

The swelling behaviors of the inserts were evaluated in IPBS (pH 7.4, 34 ± 0.2 °C) (*n* = 3). First, the inserts were weighed and placed in 20 mL scintillation glass vials containing IPBS. At specific time intervals (10, 20, and 30 min), the inserts were removed, dried using filter paper, and reweighed.

(1) $$Swelling\;ratio=\frac{Weight\;wet-Weight\;dry}{Weight\;dry}\times 100$$

#### 2.2.12. Ex Vivo Transcorneal Permeation Study

The sheep, rabbit, and pig corneas have all layers presented in the human cornea [32,33]. Sheep’s large eyeball, corneal thickness, and the presence of a Bowman’s layer would make the sheep cornea similar to that of humans [33,34,35,36]. Therefore, it is more appropriate for transcorneal studies and provides more accurate results than small laboratory animals [33,37,38,39]. Sheep eyes were obtained from a local slaughterhouse and the study was performed within 2 h. Before experimentation, the eyes were inspected for any defect. The eyes were placed in individual small beakers containing 2 mL of PBS (pH 7.4) with the corneas facing upward to prevent dehydration, and the beakers were kept in a water bath (34 ± 2.0 °C) [29,40]. The inserts or an equivalent concentration of the SNEDDS were subsequently applied, and the eyes were continuously bathed throughout the experiment (*n* = 3).

After one hour, the eyes were removed from the water bath, the inserts were collected, and the eyes were washed thoroughly. Each cornea was dissected using a scalpel and surgical scissors and subjected to extraction with 2 mL of methanol for 60 min with constant shaking. Subsequently, 1 mL was withdrawn and centrifuged at 6000 rpm for 10 min. Then, it was suitably diluted for analysis of the VOR and DIC contents by HPLC.

#### 2.2.13. Statistical Analysis

All the analyses were performed using GraphPad Prism version 8.0 (San Diego, CA, USA). The data are presented as the means ± SDs and were analyzed by two-way analysis of variance (ANOVA) followed by Tukey’s post hoc tests. A *p* value of less than 0.05 was considered to indicate statistical significance.

## 3. Results

### 3.1. Preparation of the SNEDDS and Formulations

VOR solubility in oils, cosolvents, and surfactants has been extensively studied and reported in the literature [30,41,42,43,44]. IPM has been investigated as an oil vehicle for solubilizing VOR, and the reported solubility of VOR in IPM is 14.0 ± 2.1 mg/mL. PG was also investigated as a cosolvent, in which the solubility of VOR was 36.6 ± 1.3 mg/mL. The solubility of VOR in the surfactant Tween 80 is 26.9 ± 2.1 mg/mL [28]. The choice of SNEDDS components is important for maximizing the drug payload, drug solubility, and system stability [45]. A previous study reported, after examining various proportions of the oil IPM and Tween 80:PG at ratios of 1:1 and 1:2, that the optimal SNEDDS proportion was 5:35 (5:23.33:11.67 IPM:Tween 80:PG) [28]. Therefore, in our study, we formulated an SNEDDS with a composition of 0.24% (*w*/*w*) VOR, 0.12% (*w*/*w*) DIC, 11.88% (*w*/*w*) IPM (oil), 31.55% (*w*/*w*) PG (cosolvent), and 56.21% (*w*/*w*) Tween 80 (surfactant). Afterward, the DS, PDI, and ZP of an aliquot of this mixture were measured after dilution with double-distilled water (1:100, *v*/*v*). The details for the preparation of the other formulations (F-SOL, F-EUD, and F-COM) are given in Section 2.2.2. Additionally, a piece of the insert was cut, diluted with double-distilled water and subjected to DS, PDI, and ZP measurements.

### 3.2. Assessment of DS, PDI, and ZP

The DS, PDI, and ZP results are presented in Table 1. The SNEDDS had a DS of approximately 22.5 ± 2.3 nm, which is near the DS reported in the literature [42,46]. Furthermore, the PDIs for SNEDDS and 3D-molded inserts were less than 0.32. The ZPs of SNEDDS and 3D-molded inserts were analyzed to evaluate their surface charge and stability. It is recommended that the ZP for nanoparticles should be between ± 30 mV. Adding Soluplus^®^ to the SNEDDS caused the ZP to be more negative because Soluplus^®^ has a negative charge. In addition, Compritol^®^ 888 ATO did not have any effect on the ZP, while the addition of Eudragit^®^ RS100 led to a ZP of 16.3 ± 2.4 mV because this polymer is cationic, containing 5% quaternary ammonium groups [47]. The quaternary ammonium groups might prolong the insert adhesion time by interaction with the sialic acid in mucin in the cornea and conjunctiva through ionic interactions [48].

### 3.3. Appearance, Thickness, Weight, Drug Content, and Surface pH

**Figure 1:**
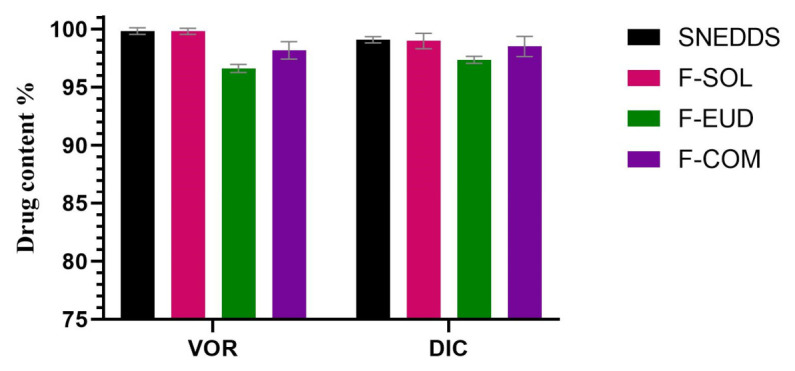
VOR and DIC content for SNEDDS and 3D-molded inserts (mean ± SD, *n* = 3).

The inserts had a smooth and uniform surface and a yellowish white–white semitransparent appearance. The weight ranged from 35.6 ± 0.8 mg to 47.6 ± 2.1 mg, and the thickness was around 1.0 mm (Table 1). Human tears have a pH from 6.5 to 7.6. Due to the buffering capacity of the eye products, the ocular globe is tolerant to topically applied formulations within a pH range of 3.0 to 8.6 [49]. The surface pH of the SNEDDS was 7.4 and it is suitable for eye administration (Table 1). The drug contents in the SNEDDS and inserts varied from 96.6 ± 0.3% to 99.8 ± 0.3% (Figure 1).

### 3.4. DSC

**Figure 2:**
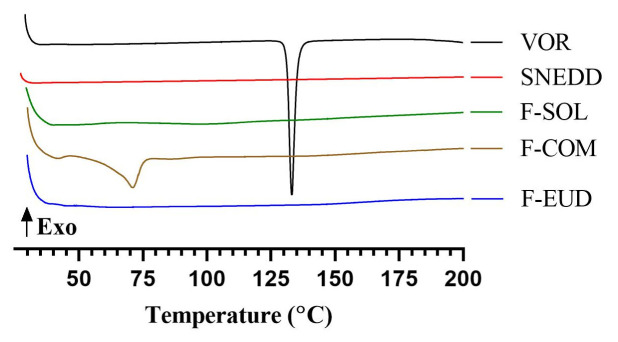
DSC thermograms for VOR, SNEDD, F-SOL, F-COM, and F-EUD inserts.

The DSC thermograms of the VOR, SNEDDS and inserts are illustrated in Figure 2. The VOR revealed a sharp endothermic peak at 134 °C, corresponding to its melting point. The characteristic peak of VOR was absent in the SNEDD. Furthermore, the addition of Soluplus^®^ and other excipients did not interact with SNEDD. The complete absence of this peak in the thermograms of all the formulations indicated that the drug was dissolved in the components of the SNEDDS [21,50]. F-COM exhibited an endothermic peak around 72 °C, and this is the melting point of Compritol^®^ 888 ATO [51]. Used polymers were reported to be stable with no degradation sign at temperature less than 200 °C [51,52,53]. Therefore, the temperature (115 °C) used for preparing the 3D-molded inserts was suitable.

### 3.5. FTIR

**Figure 3:**
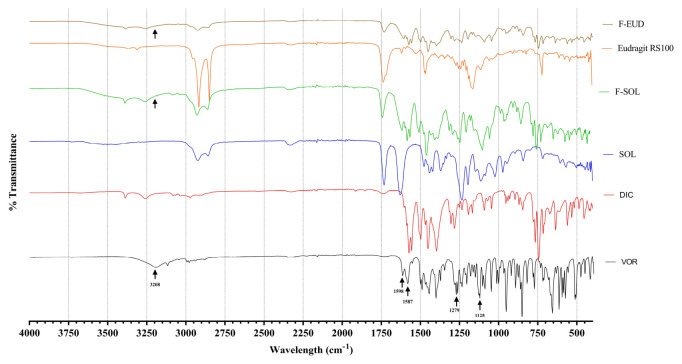
FT-IR spectra for drugs (VOR and DIC), pure polymers (SOL and EUD), and 3D-molded inserts (F-SOL and F-EUD).

FT-IR was used to examine the changes in the chemical structures of VOR and DIC. The spectrum of VOR (Figure 3) presented the following characteristic peaks: O-H stretching at 3208 cm^−1^, C-C aromatic stretching bands at 1598 cm^−1^ and 1587 cm^−1^, C-N stretching at 1279 cm^−1^, C-O stretching at 1128 cm^−1^, and aromatic banding vibrations at 856 cm^−1^, 778 cm^−1^, 667 cm^−1^ and 663 cm^−1^ [54]. The spectrum of DIC (Figure 3) presented characteristic peaks, including COOH stretching at 3310 cm^−1^, N–H stretching at 3430 cm^−1^ and C=C stretching at 1654 cm^−1^ [55]. In the FT-IR spectra of F-SOL and F-EUD, all the characteristic peaks for both 3D-molded inserts were present except for the VOR O-H stretching peak at 3208 cm^−1^. This absence might indicate the formation of hydrogen bonds between the drug and other excipients and is in agreement with literature reports [56]. A report investigated using high-HLB surfactants (Pluronic F127 and Tween 80) or low-HLB surfactants (Span 80 and Span 60) with cholesterol to formulate proniosomes loaded with voriconazole. Authors confirmed the formation of hydrogen bonding between VOR and the excipients and claimed that it enhanced the solubility and drug dissolution [50].

### 3.6. In Vitro Release

**Figure 4:**
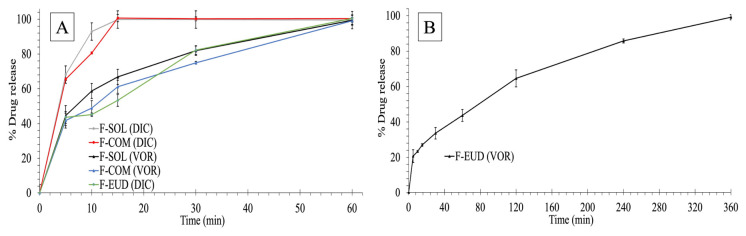
VOR and DIC in vitro release study. (**A**) inserts released drugs within 60 min. (**B**) inserts released drugs withing 360 min (mean ± SD, *n* = 3).

The in vitro release profiles of the VOR and DIC inserts are shown in Figure 4A,B. All formulations exhibited complete release of VOR and DIC within 60 min, except F-EUD, which completely released VOR within 360 min. The inserts were composed of either 100% or 75% Soluplus^®^, a hydrophilic polymer. This explains why most of the inserts exhibited complete drug release (Figure 4A). Significantly less DIC was released from F-EUD than that from other formulations within the first 15 min. However, DIC release was within 60 min. Surprisingly, F-COM, which contains Compritol^®^ 888 ATO, did not have a significant effect on the VOR and DIC release profiles compared with the effects of Soluplus^®^ (Figure 4A). Therefore, Compritol^®^ 888 ATO was not considered for further analysis.

### 3.7. Swelling Behavior

**Figure 5:**
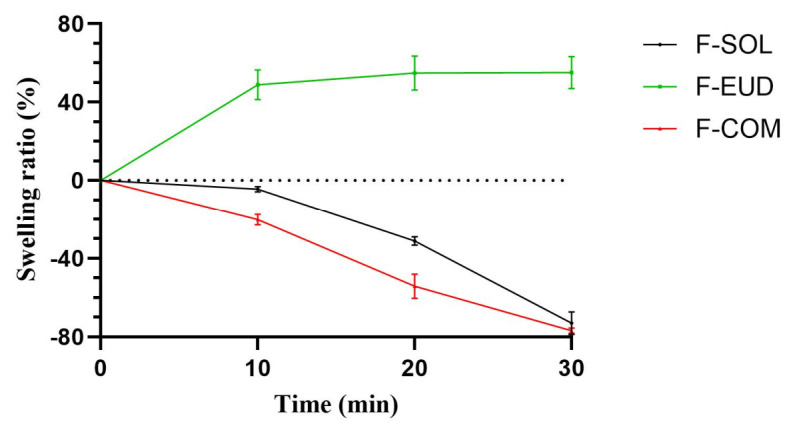
Swelling ratio (%) of 3D-molded inserts (mean ± SD, *n* = 3).

The swelling behaviors of the inserts are summarized in Figure 5. The swelling ratio shows the polymer’s capacity to absorb water [57,58]. The capacity of F-EUD was greater than that of F-SOL and F-COM because of Eudragit^®^ RS100. Eudragit^®^ RS100 is insoluble in water but absorbs water and swells in the pH range of 2–8 due to the quaternary amino groups [59]. This amino group might prolong the insert contact time by interaction with the sialic acid in mucin in the cornea and conjunctiva through ionic interactions [60]. During the experiment, F-SOL and F-COM gradually decreased in weight over time, whereas F-EUD exhibited a 50% increase in its initial weight. Soluplus^®^ is a hydrophilic polymer, which explains why F-SOL and F-COM inserts decreased in weight. On the other hand, the F-COM vial was turbid because of a water-insoluble excipient (Compritol^®^ 888 ATO), which does not swell or erode inside the aqueous media [61].

### 3.8. Ex Vivo Transcorneal Permeation Study

**Figure 6:**
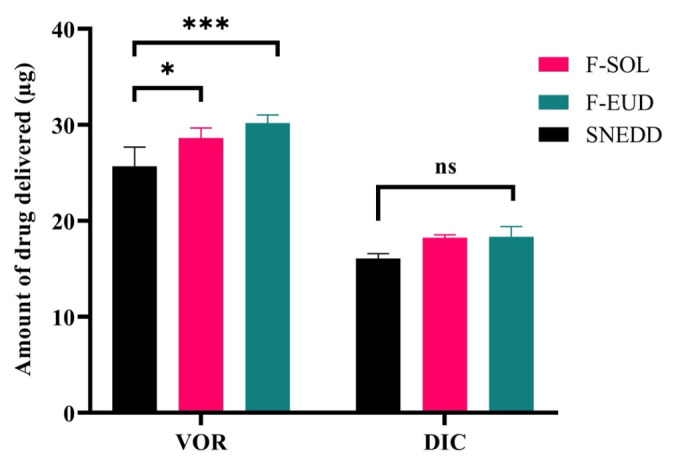
Quantities of VOR and DIC delivered to the cornea from SNEDD and leading 3D-molded inserts (mean ± SD, *n* = 3, ns = no significant, * *p* < 0.05, *** *p* < 0.001).

The permeation results of the VOR inserts and SNEDDS are illustrated in Figure 6. The SNEDDS (control) delivered an average of 25.7 ± 2.0 μg of VOR to the cornea, whereas the F-SOL and F-EUD inserts delivered averages of 28.6 ± 1.1 μg and 30.2 ± 0.9 μg of VOR to the cornea, respectively. There were significant differences between SNEDDS and F-SOL and between SNEDDS and F-EUD, with *p* values of 0.0171 and 0.0009, respectively. On the other hand, there were no significant differences in drug delivery by the DIC formulations. Furthermore, the SNEDDS delivered an average of 16.7 ± 0.5 μg of DIC to the cornea, whereas the F-SOL and F-EUD inserts delivered averages of 18.2 ± 0.3 μg and 18.3 ± 1.1 μg of DIC to the cornea, respectively.

## 4. Discussion

Increasing the proportion of oil in SNEDDS while maintaining the same surfactant concentration results in an increase in the droplet size (DS). This phenomenon is ascribed when the oil droplets join the surfactant chain, resulting in change in the surface curvature of the droplets. This results in deformation of the interfacial layer of oil droplets and the surfactant chain, causing droplet enlargement [21,28,62]. Therefore, reducing the oil percentage is crucial for reducing the DS. Another important variable that might influence the DS is the surfactant concentration. Increasing the surfactant concentration decreases the DS because the surfactant covers the oil droplets and prevents their coalescence and enlargement. Furthermore, the stability of oil globules is improved by the aggregation of the surfactant at their interfacial layer [63]. Therefore, the percentages of oil and surfactant in this project were guided by literature data, which contributed to the attainment of these promising results. Adding Soluplus^®^ and Eudragit^®^ RS100 or Compritol^®^ 888 ATO to the SNEDDS led to increases in the DS and PDI, as reported in the literature [2,19,64].

The uniformity range for a particle size distribution (PSD) was estimated by the PDI. This analysis provides an estimation on the stability of the system [65]. The system becomes more stable if it is closer to 0. If it increases to 1, which is the maximum range of PDI, the system is heterogeneous, unstable, and may lead to phase separation, which is one of the instability phases known as Ostwald ripening and coalescence [66]. Therefore, having a homogeneous PSD is important for nanoformulations, and it is known that having PDI less than 0.35 must be achieved in drug delivery systems. The PDI results were acceptable (less than 0.35).

It is recommended that the zeta potential (ZP) for nanoparticles should be around ±30 mV. This range indicates that the electrostatic repulsion between droplets is high, preventing them from aggregation and improving their stability [66]. On the other hand, the Hydrophilic-Lipophilic Balance (HLB) of IPM, PG, and Tween 80 is 11, 11.6, and 15, respectively. The solvent (IPM) and cosolvent (PG) are relatively more hydrophobic than the non-ionic surfactant (Tween 80). IPM and PG are fatty oils known for their ability to stabilize nanoformulations thermodynamically when they come into contact with an aqueous phase [67]. Additionally, the higher molecular weight and HLB of Tween 80 as a stabilizer (the non-ionic surfactant) ensure the spontaneous formation of oil-in-water stable nanoemulsion by steric hindrance that prevents particle aggregation and guarantees the long-term stability [2,68,69].

The in vitro drug release of F-EUD showed a prolonged release due to the presence of quaternary ammonium groups, which results in pH-independent release, poor solubility, and lower permeability and controls the drug release mechanism [59]. Although F-EUD maintained its structural integrity throughout the study period, which may enhance patient comfort, the swelling of Eudragit^®^ RS100 eased the diffusion of the release medium and VOR from the insert, which allowed F-EUD to exhibit highest drug release after 360 min (Figure 4A,B). The release of VOR from F-EUD displayed a biphasic pattern that was initially fast, with approximately 33.7 ± 3.3% release within the first 30 min, followed by a continuous release phase during which 99.2 ± 1.5% was released after 360 min (Figure 4B). This initial burst release could be attributed to the drug portion that might be present in Soluplus^®^, whereas the second slow release phase of VOR from the insert matrix was due to diffusion [50,70].

Nanoemulsions (SNEDDS) have the capability to improve the drug uptake and transcorneal permeation due to the presence of surfactants and cosurfactants which increase the membrane permeability. Therefore, the F-SOL and F-EUD inserts improved the transcorneal permeation and corneal deposition of VOR and DIC due to the presence of surfactants and cosurfactants [71]. However, the extent of enhancement differed between the two drugs because of their distinct physicochemical properties. VOR is a highly lipophilic drug with very low aqueous solubility. Therefore, its corneal permeation is often limited by dissolution and partitioning into the lipophilic corneal epithelium. Incorporation into SNEDDS droplets maintains voriconazole in a solubilized state and facilitates its partitioning into the epithelial layer through the large interfacial surface area generated upon nanoemulsification. In addition, the surfactants, cosurfactants and solvents present in the SNEDDS may enhance epithelial permeability, resulting in significantly improved permeation. In contrast, DIC has higher aqueous solubility than VOR. Consequently, its permeation is less dependent on solubilization enhancement. Since it is already readily available in the aqueous phase, incorporation into SNEDDS droplets may provide limited additional benefit for transcorneal transport. Moreover, the corneal epithelium facilitates the absorption of droplets smaller than 200 nm via receptor-mediated endocytosis [72]. Furthermore, covering a larger surface area with small droplets would enhance penetration of drugs [73]. The VOR minimum inhibitory concentrations (MICs) against *Candida albicans* and *Acanthamoeba castellanii* are 0.45 μg/mL and 0.5 μg/mL, respectively [74,75]. In the presence of DIC at a minimum concentration of 16 μg/mL, the MIC for VOR against *Acanthamoeba castellanii* is reduced to 0.25 μg/mL [75]. Furthermore, DIC inhibits COX and decreases the production of prostaglandins, leading to relieved swelling and pain in the eye caused by the infectious agents [76]. Therefore, incorporating DIC with VOR would improve the efficacy of VOR against these infectious agents at low doses and manage the associated pain and inflammation. In addition, the amount of VOR delivered to the cornea exceeded these reported MICs. Therefore, the SNEDDS, F-SOL insert, and F-EUD insert are promising materials for delivering VOR.

## 5. Conclusions

The developed 3D-molded inserts, loaded with a voriconazole and diclofenac SNEDDS, represent a significant step toward safer and more effective treatment for *Acanthamoeba* and mycotic keratitis. By localizing drug delivery, this approach mitigates the systemic side effects of oral or intravenous routes while enhancing patient compliance through extended release. Our formulations successfully achieved key quality attributes, including sub-100 nm droplet sizes, and demonstrated tunable release profiles—from immediate (F-SOL, F-COM) to 6 h extended release (F-EUD). Most importantly, the delivered corneal drug concentrations surpassed the established MICs for both *C. albicans* and *A. castellanii*. While further in vivo validation is a logical next step, these findings strongly support the clinical potential of our formulated inserts as a drug delivery system for treating severe corneal infections.

## Figures and Tables

**Table 1:** Appearance, weight, thickness, surface pH, droplet size, PDI, and Zeta potential for SNEDDS and 3D-molded inserts (mean ± SD, *n* = 3).

Formulation	Appearance	Weight (mg)	Thickness (mm)	Surface pH	Droplet Size (nm)	PDI	Zeta Potential (mV)
SNEDD	-	-	-	-	22.5 ± 2.3	0.1 ± 0.0	−5.3 ± 0.2
F-SOL	Yellowish white	35.6 ± 0.8	1.0 ± 0.0	7.4 ± 0.0	63.6 ± 2.2	0.3 ± 0.0	−13.8 ± 0.3
F-EUD	White	47.6 ± 2.1	1.1 ± 0.1	7.4 ± 0.0	83.8 ± 1.3	0.3 ± 0.0	16.3 ± 2.4
F-COM	White	42.2 ± 1.4	1.0 ± 0.1	7.4 ± 0.0	84.3 ± 1.1	0.2 ± 0.0	−14.6 ± 0.1

## Data Availability

The original contributions presented in this study are included in the article. Further inquiries can be directed to the corresponding author.

## References

[1] Alawfi B.S., Khan N.A., Lloyd D., Siddiqui R. (2024). *Acanthamoeba* Keratitis: New Hopes for Potential Interventions for a Curable but Often Refractory Disease. Expert Rev. Ophthalmol..

[2] Tian B., Yan Q., Wang J., Ding C., Sai S. (2017). Enhanced Antifungal Activity of Voriconazole-Loaded Nanostructured Lipid Carriers against *Candida albicans* with a Dimorphic Switching Model. Int. J. Nanomed..

[3] *Acanthamoeba* Infections|*Acanthamoeba* Infections|CDC. https://www.cdc.gov/acanthamoeba/about/index.html?CDC_AAref_Val=https://www.cdc.gov/parasites/acanthamoeba/health_profes-%252520sionals/acanthamoeba_keratitis_hcp.html.

[4] Maycock N.J.R., Jayaswal R. (2016). Update on *Acanthamoeba* Keratitis: Diagnosis, Treatment, and Outcomes. Cornea.

[5] Shing B., Balen M., McKerrow J.H., Debnath A. (2021). *Acanthamoeba* Keratitis: An Update on Amebicidal and Cysticidal Drug Screening Methodologies and Potential Treatment with Azole Drugs. Expert Rev. Anti Infect. Ther..

[6] de Lacerda A.G., Lira M. (2021). *Acanthamoeba* Keratitis: A Review of Biology, Pathophysiology and Epidemiology. Ophthalmic Physiol. Opt..

[7] Elsheikha H.M., Siddiqui R., Khan N.A. (2020). Drug Discovery against *Acanthamoeba* Infections: Present Knowledge and Unmet Needs. Pathogens.

[8] Bacterial Conjunctivitis (Pink Eye). https://emedicine.medscape.com/article/211214-overview#a4.

[9] González-Chomón C., Concheiro A., Alvarez-Lorenzo C. (2013). Soft Contact Lenses for Controlled Ocular Delivery: 50 Years in the Making. Ther. Deliv..

[10] Peng C.C., Burke M.T., Carbia B.E., Plummer C., Chauhan A. (2012). Extended Drug Delivery by Contact Lenses for Glaucoma Therapy. J. Control. Release.

[11] Rykowska I., Nowak I., Nowak R. (2021). Soft Contact Lenses as Drug Delivery Systems: A Review. Molecules.

[12] Xu J., Xue Y., Hu G., Lin T., Gou J., Yin T., He H., Zhang Y., Tang X. (2018). A Comprehensive Review on Contact Lens for Ophthalmic Drug Delivery. J. Control. Release.

[13] Quan Z., Liu X., Lu Y., Pan J., Liu C., Pan W., Li Y., Huang Y., Sun J. (2026). A Temperature-Sensitive Gel of Pranoprofen with Stable Tear Film, Anti-Inflammatory, and Sustained-Release Effects for Alleviating Dry Eye Symptoms. Exp. Eye Res..

[14] Clary R.T., Deja E., Rittmann B., Bearman G. (2025). Impact of Voriconazole Therapeutic Drug Monitoring on Adverse Effects and Clinical Outcomes: A Literature Review. Curr. Infect. Dis. Rep..

[15] Siafaka P.I., Okur N.Ü., Mone M., Giannakopoulou S., Er S., Pavlidou E., Karavas E., Bikiaris D.N. (2016). Two Different Approaches for Oral Administration of Voriconazole Loaded Formulations: Electrospun Fibers versus β-Cyclodextrin Complexes. Int. J. Mol. Sci..

[16] Sun X., Yu Z., Cai Z., Yu L., Lv Y. (2016). Voriconazole Composited Polyvinyl Alcohol/Hydroxypropyl-β-Cyclodextrin Nanofibers for Ophthalmic Delivery. PLoS ONE.

[17] Mourad A., Perfect J.R. (2018). Tolerability Profile of the Current Antifungal Armoury. J. Antimicrob. Chemother..

[18] Shettar A., Shankar V.K., Ajjarapu S., Kulkarni V.I., Repka M.A., Murthy S.N. (2021). Development and Characterization of Novel Topical Oil/PEG Creams of Voriconazole for the Treatment of Fungal Infections. J. Drug Deliv. Sci. Technol..

[19] Sanil K., Almotairy A., Uttreja P., Ashour E.A. (2024). Formulation Development and Evaluation of Cannabidiol Hot-Melt Extruded Solid Self-Emulsifying Drug Delivery System for Oral Applications. AAPS PharmSciTech.

[20] Buya A.B., Beloqui A., Memvanga P.B., Préat V. (2020). Self-Nano-Emulsifying Drug-Delivery Systems: From the Development to the Current Applications and Challenges in Oral Drug Delivery. Pharmaceutics.

[21] Ali H.S.M., Hanafy A.F., Bafail R., Alrbyawi H., Almaghrabi M., Alahmadi Y.M., El Achy S. (2024). Locally Acting Budesonide-Loaded Solid Self-Microemulsifying Drug Delivery Systems (SMEDDS) for Distal Ulcerative Colitis. Int. J. Nanomed..

[22] Guillot A.J., Petalas D., Skondra P., Rico H., Garrigues T.M., Melero A. (2021). Ciprofloxacin Self-Dissolvable Soluplus Based Polymeric Films: A Novel Proposal to Improve the Management of Eye Infections. Drug Deliv. Transl. Res..

[23] Alvarez-Rivera F., Fernández-Villanueva D., Concheiro A., Alvarez-Lorenzo C. (2016). α-Lipoic Acid in Soluplus^®^ Polymeric Nanomicelles for Ocular Treatment of Diabetes-Associated Corneal Diseases. J. Pharm. Sci..

[24] Mirzaeei S., Taghe S., Alany R.G., Nokhodchi A. (2022). Eudragit^®^ L100/Polyvinyl Alcohol Nanoparticles Impregnated Mucoadhesive Films as Ocular Inserts for Controlled Delivery of Erythromycin: Development, Characterization and In Vivo Evaluation. Biomedicines.

[25] Santos S.S., Lorenzoni A., Ferreira L.M., Mattiazzi J., Adams A.I.H., Denardi L.B., Alves S.H., Schaffazick S.R., Cruz L. (2013). Clotrimazole-Loaded Eudragit^®^ RS100 Nanocapsules: Preparation, Characterization and in Vitro Evaluation of Antifungal Activity against Candida Species. Mater. Sci. Eng. C.

[26] Aburahma M.H., Badr-Eldin S.M. (2014). Compritol 888 ATO: A Multifunctional Lipid Excipient in Drug Delivery Systems and Nanopharmaceuticals. Expert Opin. Drug Deliv..

[27] Chaves P.D.S., Frank L.A., Frank A.G., Pohlmann A.R., Guterres S.S., Beck R.C.R. (2018). Mucoadhesive Properties of Eudragit^®^RS100, Eudragit^®^S100, and Poly(ε-Caprolactone) Nanocapsules: Influence of the Vehicle and the Mucosal Surface. AAPS PharmSciTech.

[28] Kumar R., Sinha V.R. (2014). Preparation and Optimization of Voriconazole Microemulsion for Ocular Delivery. Colloids Surf. B Biointerfaces.

[29] Morgan S.R., Pilia N., Hewitt M., Moses R.L., Moseley R., Lewis P.N., Morrison P.W.J., Kelly S.L., Parker J.E., Whitaker D. (2020). Controlled in Vitro Delivery of Voriconazole and Diclofenac to the Cornea Using Contact Lenses for the Treatment of *Acanthamoeba* Keratitis. Int. J. Pharm..

[30] Rasoanirina B.N.V., Lassoued M.A., Kamoun A., Bahloul B., Miladi K., Sfar S. (2020). Voriconazole-Loaded Self-Nanoemulsifying Drug Delivery System (SNEDDS) to Improve Transcorneal Permeability. Pharm. Dev. Technol..

[31] Karnik I., Youssef A.A.A., Joshi P., Munnangi S.R., Narala S., Varner C., Vemula S.K., Majumdar S., Repka M. (2023). Formulation Development and Characterization of Dual Drug Loaded Hot-Melt Extruded Inserts for Better Ocular Therapeutic Outcomes: Sulfacetamide/Prednisolone. J. Drug Deliv. Sci. Technol..

[32] Cafaro T.A., Suarez M.F., Maldonado C., Croxatto J.O., Insfrán C., Urrets-Zavalía J.A., Serra H.M. (2015). On the Cornea of Healthy Merino Sheep: A Detailed Ex Vivo Confocal, Histological and Ultrastructural Study. Anat. Histol. Embryol..

[33] Greene C.A., Misra S.L., Lee H., McKelvie J., Kapadia K., McFarlane R., McGhee C.N.J., Green C.R., Sherwin T. (2018). The Sheep Cornea: Structural and Clinical Characteristics. Curr. Eye Res..

[34] Li H.F., Petroll W.M., Møller-Pedersen T., Maurer J.K., Cavanagh H.D., Jester J.V. (1997). Epithelial and Corneal Thickness Measurements by in Vivo Confocal Microscopy through Focusing (CMTF). Curr. Eye Res..

[35] Nautscher N., Bauer A., Steffl M., Amselgruber W.M. (2016). Comparative Morphological Evaluation of Domestic Animal Cornea. Vet. Ophthalmol..

[36] Lopinto A.J., Pirie C.G., Bedenice D., Ayres S.D.L. (2017). Corneal Thickness of Eyes of Healthy Goats, Sheep, and Alpacas Manually Measured by Use of a Portable Spectral-Domain Optical Coherence Tomography Device. Am. J. Vet. Res..

[37] De Hoon I., Boukherroub R., De Smedt S.C., Szunerits S., Sauvage F. (2023). In Vitro and Ex Vivo Models for Assessing Drug Permeation across the Cornea. Mol. Pharm..

[38] Kanellopoulos A.J., Asimellis G. (2013). In Vivo Three-Dimensional Corneal Epithelium Imaging in Normal Eyes by Anterior-Segment Optical Coherence Tomography: A Clinical Reference Study. Cornea.

[39] Viehmeister K., Manuelli A., Guerin C., Kappes S., Lamprecht A. (2024). Imaging-Based Drug Penetration Profiling in an Excised Sheep Cornea Model. Pharmaceutics.

[40] Hewitt M.G., Morrison P.W.J., Boostrom H.M., Morgan S.R., Fallon M., Lewis P.N., Whitaker D., Brancale A., Varricchio C., Quantock A.J. (2020). In Vitro Topical Delivery of Chlorhexidine to the Cornea: Enhancement Using Drug-Loaded Contact Lenses and β-Cyclodextrin Complexation, and the Importance of Simulating Tear Irrigation. Mol. Pharm..

[41] Bahloul B., Lassoued M.A., Sfar S. (2014). A Novel Approach for the Development and Optimization of Self Emulsifying Drug Delivery System Using HLB and Response Surface Methodology: Application to Fenofibrate Encapsulation. Int. J. Pharm..

[42] Rasoanirina B.N.V., Lassoued M.A., Miladi K., Razafindrakoto Z., Chaâbane-Banaoues R., Ramanitrahasimbola D., Cornet M., Sfar S. (2020). Self-Nanoemulsifying Drug Delivery System to Improve Transcorneal Permeability of Voriconazole: In-Vivo Studies. J. Pharm. Pharmacol..

[43] Raju Y.P., Hyndavi N., Chowdary V.H., Nair R.S., Basha D.J., Tejeswari N. (2017). In Vitro Assessment of Non-Irritant Microemulsified Voriconazole Hydrogel System. Artif. Cells Nanomed. Biotechnol..

[44] Paliwal H., Prajapati B.G. (2025). Development and In Vitro Characterization of a Voriconazole Loaded Nanoemulsion for Potential Intranasal Drug Delivery. Bionanoscience.

[45] Yousry C., Zikry P.M., Basalious E.B., El-Gazayerly O.N. (2020). Self-Nanoemulsifying System Optimization for Higher Terconazole Solubilization and Non-Irritant Ocular Administration. Adv. Pharm. Bull..

[46] Elbahwy I.A., Lupo N., Ibrahim H.M., Ismael H.R., Kasem A.A., Caliskan C., Matuszczak B., Bernkop-Schnürch A. (2018). Mucoadhesive Self-Emulsifying Delivery Systems for Ocular Administration of Econazole. Int. J. Pharm..

[47] Rowe R.C., Sheskey P.J., Quinn M.E. (2009). Handbook of Pharmaceutical Excipients.

[48] Santos G., Delgado E., Silva B., Braz B.S., Gonçalves L. (2025). Topical Ocular Drug Delivery: The Impact of Permeation Enhancers. Pharmaceutics.

[49] Bhawale R., Srivastava V., Mehra N.K., Jain K., Yadav A.K. (2025). Advances in Ophthalmic Formulation Development. Advances in Pharmaceutical Product Development.

[50] El-Emam G.A., Girgis G.N.S., El Sokkary M.M.A., El-Azeem Soliman O.A., Abd El Gawad A.E.G.H. (2020). Ocular Inserts of Voriconazole-Loaded Proniosomal Gels: Formulation, Evaluation and Microbiological Studies. Int. J. Nanomed..

[51] Hippalgaonkar K., Adelli G.R., Hippalgaonkar K., Repka M.A., Majumdar S. (2013). Indomethacin-Loaded Solid Lipid Nanoparticles for Ocular Delivery: Development, Characterization, and in Vitro Evaluation. J. Ocul. Pharmacol. Ther..

[52] Raval M.K., Patel J.M., Parikh R.K., Sheth N.R. (2014). Studies on Influence of Polymers and Excipients on Crystallization Behavior of Metformin HCl to Improve the Manufacturability. Part. Sci. Technol..

[53] Feng S., Zhang Z., Almotairy A., Repka M.A. (2023). Development and Evaluation of Polymeric Mixed Micelles Prepared Using Hot-Melt Extrusion for Extended Delivery of Poorly Water-Soluble Drugs. J. Pharm. Sci..

[54] Füredi P., Pápay Z.E., Kovács K., Kiss B.D., Ludányi K., Antal I., Klebovich I. (2017). Development and Characterization of the Voriconazole Loaded Lipid-Based Nanoparticles. J. Pharm. Biomed. Anal..

[55] Suhail M., Khan A., Rosenholm J.M., Minhas M.U., Wu P.C. (2021). Fabrication and Characterization of Diclofenac Sodium Loaded Hydrogels of Sodium Alginate as Sustained Release Carrier. Gels.

[56] Madni A., Rahim M.A., Mahmood M.A., Jabar A., Rehman M., Shah H., Khan A., Tahir N., Shah A. (2018). Enhancement of Dissolution and Skin Permeability of Pentazocine by Proniosomes and Niosomal Gel. AAPS PharmSciTech.

[57] Sharma R., Kamboj S., Singh G., Rana V. (2016). Development of Aprepitant Loaded Orally Disintegrating Films for Enhanced Pharmacokinetic Performance. Eur. J. Pharm. Sci..

[58] Almotairy A., Alyahya M., Althobaiti A., Almutairi M., Bandari S., Ashour E.A., Repka M.A. (2023). Disulfiram 3D Printed Film Produced via Hot-Melt Extrusion Techniques as a Potential Anticervical Cancer Candidate. Int. J. Pharm..

[59] Jain S.K., Jain A.K., Rajpoot K. (2020). Expedition of Eudragit^®^ Polymers in the Development of Novel Drug Delivery Systems. Curr. Drug Deliv..

[60] Mandal B., Alexander K.S., Riga A.T. (2010). Sulfacetamide Loaded Eudragit RL100 Nanosuspension with Potential for Ocular Delivery. J. Pharm. Pharm. Sci..

[61] Kreye F., Siepmann F., Willart J.F., Descamps M., Siepmann J. (2011). Drug Release Mechanisms of Cast Lipid Implants. Eur. J. Pharm. Biopharm..

[62] Shanmugam S., Baskaran R., Balakrishnan P., Thapa P., Yong C.S., Yoo B.K. (2011). Solid Self-Nanoemulsifying Drug Delivery System (S-SNEDDS) Containing Phosphatidylcholine for Enhanced Bioavailability of Highly Lipophilic Bioactive Carotenoid Lutein. Eur. J. Pharm. Biopharm..

[63] Lee D.W., Marasini N., Poudel B.K., Kim J.H., Cho H.J., Moon B.K., Choi H.G., Yong C.S., Kim J.O. (2014). Application of Box–Behnken Design in the Preparation and Optimization of Fenofibrate-Loaded Self-Microemulsifying Drug Delivery System (SMEDDS). J. Microencapsul..

[64] Tayel S.A., El-Nabarawi M.A., Tadros M.I., Abd-Elsalam W.H. (2013). Positively Charged Polymeric Nanoparticle Reservoirs of Terbinafine Hydrochloride: Preclinical Implications for Controlled Drug Delivery in the Aqueous Humor of Rabbits. AAPS PharmSciTech.

[65] Jiang Y., Zhang C., Yuan J., Wu Y., Li F., Li D., Huang Q. (2019). Effects of Pectin Polydispersity on Zein/Pectin Composite Nanoparticles (ZAPs) as High Internal-Phase Pickering Emulsion Stabilizers. Carbohydr. Polym..

[66] Laxmi M., Bhardwaj A., Mehta S., Mehta A. (2015). Development and Characterization of Nanoemulsion as Carrier for the Enhancement of Bioavailability of Artemether. Artif. Cells Nanomed. Biotechnol..

[67] Garti N., Aserin A., Tiunova I., Fanun M. (2000). A DSC Study of Water Behavior in Water-in-Oil Microemulsions Stabilized by Sucrose Esters and Butanol. Colloids Surf. A Physicochem. Eng. Asp..

[68] Schwarz J.C., Klang V., Hoppel M., Mahrhauser D., Valenta C. (2012). Natural Microemulsions: Formulation Design and Skin Interaction. Eur. J. Pharm. Biopharm..

[69] Mishra P.R., Al Shaal L., Müller R.H., Keck C.M. (2009). Production and Characterization of Hesperetin Nanosuspensions for Dermal Delivery. Int. J. Pharm..

[70] Akhtar N., Kumar Singh R., Pathak K. (2017). Exploring the Potential of Complex-Vesicle Based Niosomal Ocular System Loaded with Azithromycin: Development of in Situ Gel and Ex Vivo Characterization. Pharm. Biomed. Res..

[71] Ammar H.O., Salama H.A., Ghorab M., Mahmoud A.A. (2009). Nanoemulsion as a Potential Ophthalmic Delivery System for Dorzolamide Hydrochloride. AAPS PharmSciTech.

[72] Sita V.G., Vavia P. (2020). Bromocriptine Nanoemulsion-Loaded Transdermal Gel: Optimization Using Factorial Design, In Vitro and In Vivo Evaluation. AAPS PharmSciTech.

[73] Shaker D.S., Ishak R.A.H., Ghoneim A., Elhuoni M.A. (2019). Nanoemulsion: A Review on Mechanisms for the Transdermal Delivery of Hydrophobic and Hydrophilic Drugs. Sci. Pharm..

[74] Pfaller M.A., Diekema D.J., Rex J.H., Espinel-Ingroff A., Johnson E.M., Andes D., Chaturvedi V., Ghannoum M.A., Odds F.C., Rinaldi M.G. (2006). Correlation of MIC with Outcome for Candida Species Tested against Voriconazole: Analysis and Proposal for Interpretive Breakpoints. J. Clin. Microbiol..

[75] Lamb D.C., Warrilow A.G.S., Rolley N.J., Parker J.E., Nes W.D., Smith S.N., Kelly D.E., Kelly S.L. (2015). Azole Antifungal Agents to Treat the Human Pathogens *Acanthamoeba* Castellanii and *Acanthamoeba* Polyphaga through Inhibition of Sterol 14α-Demethylase (CYP51). Antimicrob. Agents Chemother..

[76] Hersh P.S., Rice B.A., Baer J.C., Wells P.A., Lynch S.E., Mcguigan L.J.B., Foster C.S. (1990). Topical Nonsteroidal Agents and Corneal Wound Healing. Arch. Ophthalmol..

